# Complete genome analysis of *Jeotgalibacillus* sp. 10bf12, a bacterium isolated from the surface of Daihai Lake, Inner Mongolia

**DOI:** 10.1128/mra.00312-26

**Published:** 2026-05-29

**Authors:** Yanxing Wang, Qiuqin Wei, Bo Yuan, Chunling Cao, Gen Che, Lijia Ye, Yu Hong, Kai Jiang

**Affiliations:** 1 College of Life Science and Technology, Inner Mongolia Normal University 71203 , Hohhot, Inner Mongolia, China; 2 Key Laboratory of Biodiversity Conservation and Sustainable Utilization in Mongolian Plateau for College and University of Inner Mongolia Autonomous Region, Hohhot, China; 3 Department of Agriculture and Animal Husbandry of Inner Mongolia, Hohhot, Inner Mongolia, China; Nanchang University, Nanchang, Jiangxi, China

**Keywords:** *Jeotgalibacillus genus*, Daihai Lake, complete genome, 16S rRNA gene

## Abstract

This paper reports the isolation of strain 10bf12 from Daihai Lake. The complete genome of strain 10bf12 is 3,452,620 bp in size, with a G + C content of 42.59%. Cultivation experiments revealed that strain 10bf12 is adaptable to high-salinity environments with potential industrial value.

## ANNOUNCEMENT

*Jeotgalibacillus*, first described by Jung-Hoon Yoon and Norbert Weiss in 2001, has the type strain *J. alimentarius* YKJ-13ᵀ, which was isolated from traditional Korean fermented seafood, jeotgal (1). The genus comprises rod-shaped, endospore-forming bacteria; most are motile by monopolar, subpolar, or peritrichous flagella, except *J. alkaliphilus* (2). Ten validly published species are mostly isolated from saline environments, with a few (e.g., strain JSM 081008^T^) from non-saline forest soils (1–9). Their salt tolerance suggests potential for salt-tolerant wastewater treatment.

The sampling site was at 112°39′0″E, 40°36′0″N. Water temperature was 17°C; pH was 9.0; and salinity was 1.2%. Surface water and sediment were collected from different sites along Daihai Lake shore using sterilized tools. The mixture was serially diluted with sterile lake water. The 10⁻¹ dilution was filtered through a 0.22 μm membrane, and the filtrate was used as the inoculum. An aliquot of 100 μL of the inoculum was spread onto six media (LBM, LBM/10, LBM/100, LBM-10%, LBM-20%, LD). After incubation at room temperature (22°C) for 1 month, distinct colonies were purified via the streak plate method. Strain 10bf12 was finally isolated on medium containing 100 g/L NaCl.

The strain 10bf12 was inoculated into Luria-Bertani medium containing 10% NaCl and cultured to the logarithmic phase. High-quality genomic DNA was extracted using the Blood & Cell Culture DNA Kit (QIAGEN, Germany). DNA purity was assessed with a NanoDrop One spectrophotometer, and DNA concentration was precisely quantified using a Qubit 3.0 fluorometer. The DNA was repaired and adapter-ligated using the SQK-LSK109 Kit, and the library was loaded onto a prepared Nanopore PromethION flow cell for single-molecule sequencing, generating raw sequencing data (10). Base calling was performed using Guppy v3.1.5 with its super-accurate model, and reads were converted to FASTQ format. Adapters, erroneous sequences, and low-quality reads (Q ≥ 7) were filtered out, yielding qualified reads for assembly. Assembly was performed using Flye v2.7 (parameters: --plasmids --nano-raw) (11, 12). NGS was conducted on an Illumina NovaSeq platform with the Truseq Nano DNA HT Sample Preparation Kit. The assembly was polished using Pilon v1.23 with NGS data (13) and Racon v1.4.13 with ONT data (14). A custom script was used for genome circularization and trimming of redundant regions, followed by normalization with Circlator v1.5.1 (using the fixstart parameter) (15) to reposition the replication origin. The complete bacterial genome was annotated using the NCBI Prokaryotic Genome Annotation Pipeline. A phylogenetic tree was constructed using MEGA v12.0 with the maximum likelihood method (16, 17); all parameters were set to default unless otherwise specified.

**Fig 1:**
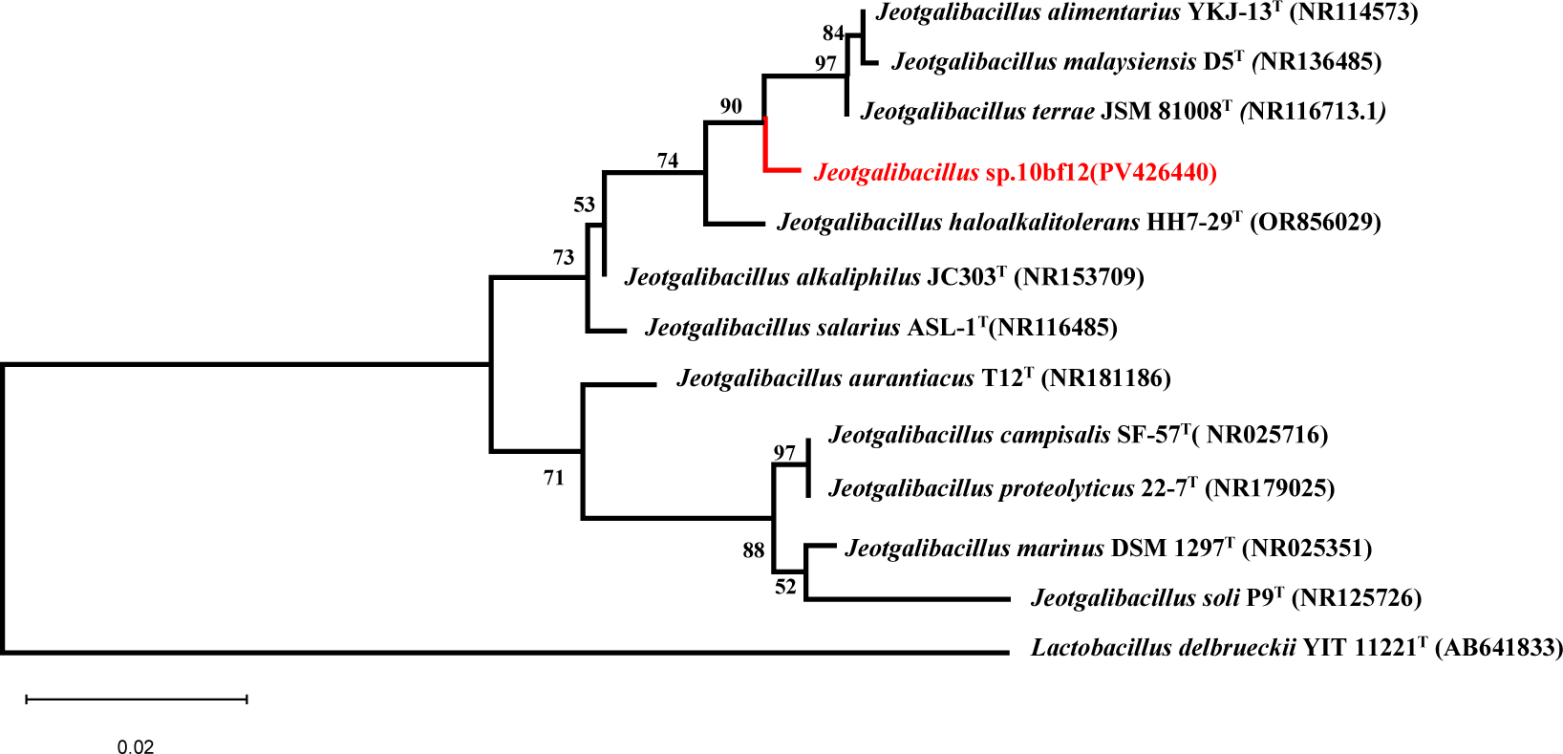
Maximum-likelihood tree based on 16S rRNA gene sequences using the Kimura two-parameter method, showing the phylogenetic position of strain 10bf12 within related genera. Bootstrap values were based on 1,000 replicates (values ≥ 50% were shown). *Lactobacillus delbrueckii* YIT 11221^T^ was used as the out-group. Bar, 0.02 changes per nucleotide position.

After sequencing strain 10bf12 in this study, a total of 1.545 Gb raw data were generated. Following reassembly, a single complete contig of 3,452,620 bp was ultimately generated, with a coverage depth exceeding 409×. The G + C content of the genomic DNA was 42.59%. Genomic data, assembly, and annotation information for strain 10bf12 are summarized in Table 1. The length of the 16S rRNA gene was 1,535 bp, and the 16S rRNA-based phylogenetic tree indicated that strain 10bf12 belongs to the genus *Jeotgalibacillus* (Fig. 1).

**TABLE 1:** Genomic data, assembly, and annotation information of strain 10bf12

Project	ONT	NGS
Raw reads	1,545,156,175 bp	1,100,000,000 bp
Filtered reads	1,476,764,324 bp	1,100,000,000 bp
Read number	87,927	
Reads N50	26,146 bp	
Longest reads	121,136 bp	
Genome size	3,452,620 bp	
Q20	–^*a*^	98.3%
Q30	–	94.56%
Genomic DNA G + C content	42.59%	42.7%
Contig number	1	
Sequencing depth (×)	409.49	
CDS	3,535	
tRNA	79	
rRNA	27	

^*a*^ –, no data available.

## Data Availability

The whole-genome data of *Jeotgalibacillus* sp. 10bf12 can be obtained under the accession CP200276 in GenBank. The 16S rRNA gene sequence has been deposited in GenBank, and the accession number is PV426440. The Sequence Read Archive (SRA) accession numbers for raw reads are SRR33097660 (NGS) and SRR33097661 (ONT). The BioSample and BioProject accession numbers are SAMN47742805 and PRJNA648636, respectively.
